# SeXX Matters in Multiple Sclerosis

**DOI:** 10.3389/fneur.2020.00616

**Published:** 2020-07-03

**Authors:** Francesca Gilli, Krista D. DiSano, Andrew R. Pachner

**Affiliations:** Department of Neurology, Dartmouth Hitchcock Medical Center, Geisel School of Medicine at Dartmouth, Lebanon, NH, United States

**Keywords:** multiple sclerosis, sex dimorphism, sex chromosome, sex hormones, neurodegeneration

## Abstract

Multiple sclerosis (MS) is the most common chronic inflammatory and neurodegenerative disease of the central nervous system (CNS). An interesting feature that this debilitating disease shares with many other inflammatory disorders is that susceptibility is higher in females than in males, with the risk of MS being three times higher in women compared to men. Nonetheless, while men have a decreased risk of developing MS, many studies suggest that males have a worse clinical outcome. MS exhibits an apparent sexual dimorphism in both the immune response and the pathophysiology of the CNS damage, ultimately affecting disease susceptibility and progression differently. Overall, women are predisposed to higher rates of inflammatory relapses than men, but men are more likely to manifest signs of disease progression and worse CNS damage. The observed sexual dimorphism in MS may be due to sex hormones and sex chromosomes, acting in parallel or combination. In this review, we outline current knowledge on the sexual dimorphism in MS and discuss the interplay of sex chromosomes, sex hormones, and the immune system in driving MS disease susceptibility and progression.

## Introduction

Multiple sclerosis (MS) is a chronic inflammatory and neurodegenerative disease of the central nervous system (CNS) in which relentless demyelination and neuroaxonal loss are the major cause of irreversible disability (1, 2). The cause of MS is still obscure, but currently the most accredited hypothesis is that a dysfunctional (overactive) immune system may result in the activation of cross-reactive immune cells to myelin and oligodendrocyte antigens (3–6). CD4^+^ T helper 1 (Th1) and Th17 cells have been principally implicated in MS pathogenesis. However, many other cell types, including CD8^+^ T cells, B cells, monocytes, neutrophils, macrophages, and microglia, have also been proposed as heavily involved in MS pathogenesis (7–12).

MS typically follows either a relapsing-remitting or a progressive disease pattern (13). Relapsing-remitting MS (RRMS) is characterized by recurrent clinical exacerbations caused by focal inflammatory and demyelinating lesions in the CNS. RRMS is the most common type of MS, although up to 65% of these patients eventually transition to a secondary progressive course (SPMS). Approximately 15% of people with MS are diagnosed with progressive MS from the onset, in the form of the disease known as primary progressive MS (PPMS). Both PPMS and SPMS are identified by steadily worsening neurologic functions thought to reflect ongoing neurodegenerative processes (14, 15).

**Figure 1:**
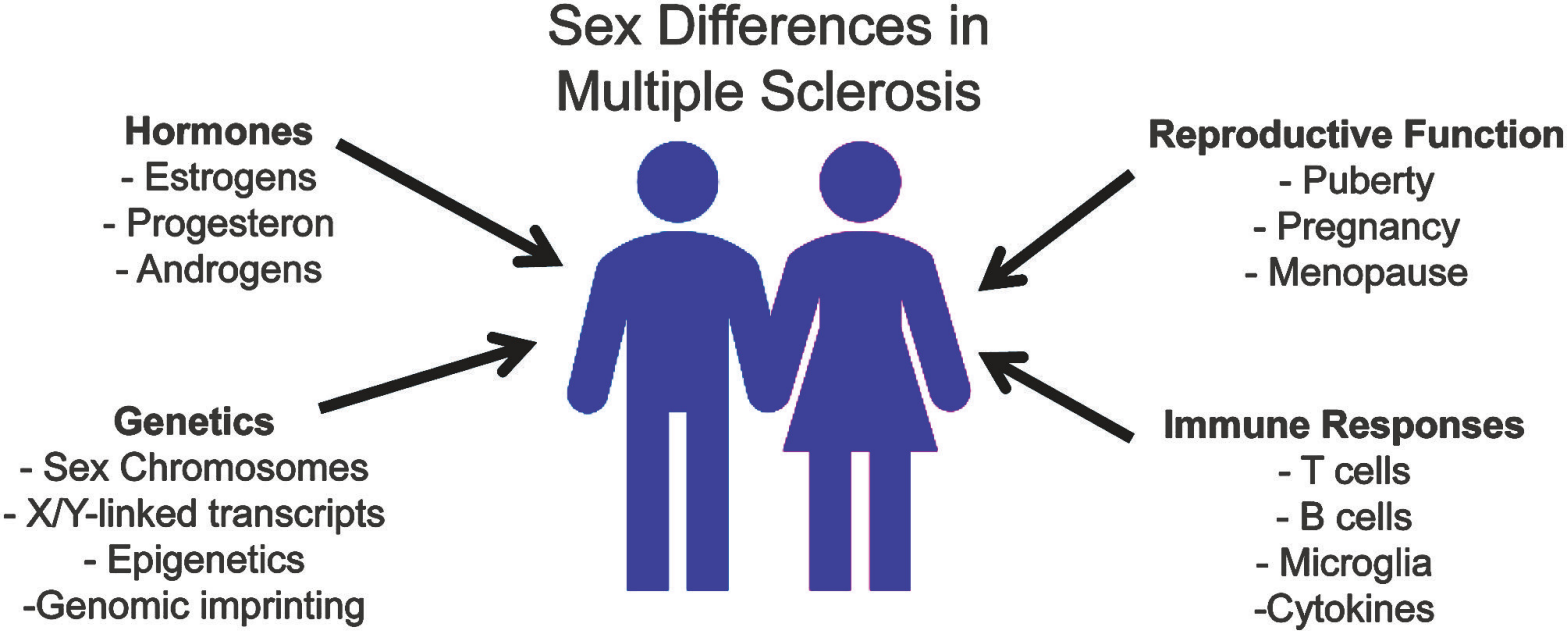
Mechanisms of sexual dimorphism that underlie sex differences in immune responses, ultimately affecting MS disease susceptibility and progression. Research regarding sex hormones has extensively been analyzed due to the pre-existing knowledge that hormone receptors affect immune cells. The three primary sex hormones that have been shown to affect the immune system are estrogen, progesterone, and testosterone. All of these hormones have also been shown to significantly affect both MS disease susceptibility and clinical outcome. Sex chromosome complement exerts its effect in promoting sexual dimorphism in MS independent of sex hormones. X-dosage compensation and escape from X-inactivation influence differential gene expression of innate and adaptive immune molecules. Y chromosome contributions include Y-linked potentially dysregulated immunity loci. Studies evaluating sexual dimorphism in immune responses focus on the interdependence of these factors, as well as their independent contributions.

In MS, the influence of biological sex has been reported on incidence, prevalence, disease course, severity, and prognosis (1, 16). While MS disproportionally affects more women than men (1, 16, 17), women more frequently present with a more benign course, with predominantly sensory symptoms, and fewer inflammatory relapses (1). In contrast, men experience more motor symptoms, cognitive impairment, and overall worse progression (18). As is the case with other immune-mediated diseases, the precise basis of the sex bias in MS is complex. It potentially involves sex hormones and reproductive history, sex-related differences in immune responsiveness, and sex-linked genetic and epigenetic factors [Figure 1; (16, 19–25)].

Current knowledge suggests that the study of the biology of sex bias may provide valuable insight into pathogenesis and mechanisms of disease progression in MS. Moreover, the effects of sex on the clinical expression of MS may be relevant to the management and treatment of patients. This review summarizes and evaluates findings from clinical and pre-clinical studies investigating the impact of biological sex on MS susceptibility, inflammatory activity, and disease progression. The aim is to provide neurologists and scientists with a clearer understanding of how sex differences affect disease development and progression and to explore how sex differences can influence the evolution of new therapeutic options for this debilitating disease.

## Clinical and Radiological Evidence of Sexual Dimorphism in Multiple Sclerosis

It is common knowledge that females and males differ in many morphological, physiological, and behavioral features. This sexual dimorphism also encompasses a wide range of disorders, including immune-mediated (20, 26), and neurodegenerative diseases (27–29).

The influence of the biological sex on MS has been demonstrated in all aspects of the disease, from increased susceptibility in women to worsened disease outcomes in men. The current section specifically focuses on the sex differences observed in disease susceptibility as well as clinical and radiological outcome measures in MS. The overview given in Table 1 suggests a more inflammatory phenotype of the disease in female patients vs. a more neurodegenerative phenotype in male patients.

**Table 1:** Sex differences in prevalence, clinical outcomes, and radiological measures in MS.

	**Females**	**Males**	**References**
Prevalence	Higher prevalence of RRMS and SPMS Lower prevalence of PPMS.	Men slightly more affected by PPMS	(30, 31)
	Earlier onset (age 20–40)	Slight preponderance in patients > age 50	(31, 32)
Clinical outcome	Slower disease progression	Higher risk of worsening when relapse-free	(33–35)
	Higher inflammatory activity (relapses)		(33, 35, 36)
		Poorer clinical outcome	(25, 36–43)
		Faster conversion from RRMS to SPMS	(31, 44–46)
Radiological measures	Inflammatory phenotype, i.e., Gd+ lesions		(47–50)
		Neurodegenerative Phenotype, i.e., hypointense T1 lesions	(47, 51)
	Higher WM atrophy	Higher GM atrophy, global brain atrophy, and cortical thickness loss with age. More cortical lesions and a faster rate of microstructural changes in WM chronic lesions.	(52–57)

### Clinical Differences in Multiple Sclerosis

Women have, on average, a two to four times higher risk of developing MS than men (58), with a constant increase of the women-to-man (w/m) ratio over time (59–61). This rapid increase observed over the last century likely reflects changes in the lifestyle, environment, or nutrition. Dietary intake, hormonal therapies, reproductive history, drinking, and smoking habits have all dramatically changed in the last century, impacting women differently from men (62). These factors are all known to influence MS risk and therefore represent potential candidates for the increasing MS susceptibility among women (63–67). Additionally, a recent age-period-cohort analysis in Norway found that social factors varying throughout the years, e.g., improved access to MS diagnosis among females, may also explain the increasing w/m ratio (68).

The female preponderance is observed in all phenotypes of the disease except for PPMS, in which men seem to be slightly more affected than women (30, 31). The rate at which patients reach SPMS is also possibly influenced by sex, with the male sex being mostly associated with earlier conversion from RRMS to SPMS (31, 44–46). The w/m ratio also generally declines with increasing age at onset, showing a female preponderance in RRMS patients between 20 and 40 years of age but a slight male predominance in patients over 50 years of age (31, 32). Hence, as a whole, women show earlier onset of the disease, significantly higher prevalence of RRMS, a slower disease progression, and a somewhat lower prevalence of PPMS.

In contrast to the clear sexual dimorphism in MS prevalence, the role of sex in influencing clinical features is less obvious. Women overall show higher inflammatory activity and less disability progression than men (33–35). Women with MS have about 20% more clinical relapses than men (36), as well as a higher relapse rate throughout the disease (33). Conversely, many natural history studies in MS suggest that male sex is generally associated with a worse clinical outcome (25, 36, 69). Men have on average a more progressive and severe disease course (37, 38), a faster time to progression (18, 44), and more significant disability over time (36, 39, 46, 69). Men tend to worsen more in relapse-free study intervals than women, and clinical relapses do not seem to influence their sustained Expanded Disability Status Scale (EDSS) change over time (35). Male sex also associates with increased occurrence of cerebellar symptoms (40) and higher incidence and severity of cognitive deficits (39, 41, 42).

Although socio-demographics may play a role in physical and cognitive disability outcomes, a study comparing disability progression between men and women with MS found increased disability among men, even after adjustment for socio-demographic factors and disease-modifying drugs (DMTs) (70). Likewise, a recent study on a large cohort of MS patients demonstrated that male sex, independent of any additional demographic or psychiatric component, represents a critical risk factor for the development of cognitive impairment (43).

Studies have additionally explored whether women with MS respond differently to DMTs than men. Current treatment of MS consists mainly of anti-inflammatory DMTs, which substantially reduce inflammatory disease activity and should also slow down disease progression. Hypothetically women might be more responsive to these anti-inflammatory therapies, ultimately resulting in the development of a less progressive disease course in females compared with males. However, currently, there is no indication of any sex-differential effect of DMTs in men or women with MS (71, 72).

### Radiological Differences in Multiple Sclerosis

Sex differences in clinical presentation, severity, and outcome of MS are also suggested by average group differences on a variety of magnetic resonance imaging (MRI) measures (38, 47, 48, 73), although this relationship has not always been confirmed (52, 74–77).

Generally, women present with a higher number of gadolinium (Gd)-enhancing lesions (47–50) indicative of a more inflammatory phenotype. Concurrently, men tend to develop more destructive lesions, i.e., hypointense T1 lesions, suggesting a more neurodegenerative phenotype in the male population (47, 51).

Regarding brain atrophy, white matter (WM) atrophy is, on average, higher in women compared to men with MS (52), whereas both global brain atrophy (53, 54) and gray matter (GM) atrophy are higher in men compared to their female counterparts (52–54). Overall, GM atrophy in men with MS is dominated by a more substantial amount of deep GM atrophy (54), and it is correlated with a worse impaired cognition (53). Furthermore, men show, on average, more cortical lesions than women in all MS phenotypes (52, 55) as well as a higher cortical thickness loss with aging compared to women (56). However, it should also be pointed out that conflicting results have been provided regarding the progression of GM volume loss over time in men and women with MS (78, 79).

Magnetization transfer imaging (MTR) studies have shown no significant differences in submicroscopic diffuse WM and GM damage between men and women with MS (52). On the other hand, using diffusion tension imaging (DTI), WM diffuse, and regional damage, especially in the thalamus, was higher in men compared to clinically matched women and controls of both sexes also correlating with a faster worsening of cognitive functions (54). DTI also demonstrated a faster rate of microstructural changes in WM chronic lesions of men with MS. Specifically, a faster increase in both parallel and perpendicular diffusivity was shown in men with MS over 12 months of follow-up. In contrast, women demonstrated much smaller changes in lesional diffusivity over time (57).

The different patterns of sex hormones may provide a possible explanation for these radiological differences in men and women across their life span. Early conventional MRI studies have shown a correlation between sex hormone levels and disease activity measured as Gd-enhancing lesions and both T1 and T2 lesions in RRMS patients of both sexes (49, 73, 80). For example, both male and female MS patients with high estradiol and low progesterone levels show, on average, more Gd-enhancing lesions compared to patients presenting with low levels of these female sex hormones (73, 80). These hormones may act in concert for synergistic immune effects, with low estradiol to progesterone ratio exerting a protective effect on MS disease activity. Notably, during the third trimester of pregnancy, i.e., when clinical and MRI disease activity is suppressed (81–84), the progesterone/estradiol ratio appears low to reduce the risks of an adverse pregnancy outcome (85, 86). Likewise, a negative correlation was observed between concentrations of the male hormone testosterone and both tissue damage as detected on MRI and neurological impairment (49, 87–89). Notably, the lowest levels of testosterone are found in patients with higher numbers of Gd-enhancing lesions (49).

The picture emerging highlights the differing MS disease features between men and women. Although MS is more prevalent and inflammatory in women, when men develop the disease, it tends to be worse, being associated with a faster clinical progression and greater disability.

## Biology of Sex Differences in Multiple Sclerosis

Sexual dimorphism consists of a phenotypic distinction between males and females of the same species and is most evident as differences in outward appearance, i.e., external morphology. Sexual dimorphism may also occur in internal organs and a wide range of biological functions, including several aspects of immunity. This section focuses on the effects of sexual dimorphism on the pathology and immunology of MS, as well as those sex-determining factors, i.e., sex hormones and chromosomes, influencing the degree of sexual size dimorphism in MS. Results from studies related to these research topics are summarized in Tables 2–4.

**Table 2:** Biology of sex differences in MS: histopathology and immune responsiveness.

	**Females**	**Males**	**References**
Histopathology		Neurodegenerative phenotype, i.e., chronic active lesions; axonal damage	(90–92)
	Slightly more pronounced remyelination		(93)
Immune responsiveness	More robust peripheral immune system response		(19, 94–105)
		Stronger neuroinflammation	(106–108)

**Table 3A:** Biology of sex differences in MS: sex hormones in males.

	**Males**		**References**
Sex hormones	Low testosterone	Worse Disability	(49, 89, 109, 110)
		More cognitive decline	(34, 39, 89)
		Enhanced risk of developing MS	(111)
	High testosterone	Protective in the development of MS	(112–115)
		Neuroprotective actions	(114, 116, 117)

**Table 3B:** Biology of sex differences in MS: sex hormones in females.

	**Females**		**References**
Sex hormones	High progesterone and estrogens	Protective effects	(118–121)
		Neuroprotective actions	(122–124)
		Reduction in Relapse Rate during pregnancy	(81, 84, 125, 126)
	Low estrogens	Higher risk of developing MS	(127)
		Fewer relapses and faster disease progression	(128, 129)
		Higher risk of developing neurodegenerative diseases, including progressive MS, during menopause	(128–131)
		Resurgence of relapses after delivery	(82, 132)

**Table 4:** Biology of sex differences in MS: sex chromosomes.

Sex chromosomes	Role of the X chromosome	Increased EAE disease severity in XX mice	(133)
		Susceptibility genes on the X chromosome	(134–138)
		Abnormalities on the X chromosome, i.e., translocation and deletions	(139)
	Role of the Y chromosome	Susceptibility genes on the Y chromosome	(140, 141)

### Sex-Related Differences in Histopathology

Not many pathological studies have evaluated sex bias in MS so far and the few studies that have mostly provided conflicting results.

By analyzing neocortical lesions and cortical thickness in the brain tissue obtained from 22 MS patients, an early study showed no differences in number, type, or distribution of cortical lesions between women and men (142). However, a more recent study performed on 182 MS brain donors and a total of 7562 analyzed lesions, showed that male patients have a higher incidence of cortical GM lesions compared to females (90), ultimately confirming previous MRI studies as described above (52, 55). The latter study also demonstrated a higher proportion of chronic active lesions in the male population (90). Similarly, in a previous study, these same lesions were reported to be increased in men with MS, although this finding was only restricted to a 45–55 age group (91).

In MS, chronic active lesions are characterized by a hypocellular demyelinated core and a hypercellular edge of activated microglia related to smoldering inflammation and axonal degeneration (143). Patients with a more severe disease have, on average, higher lesion loads compared to patients with a less severe disease course (90, 144). Chronic active lesions are also more prominent in progressive MS compared to relapsing forms of the disease. Therefore, these findings further support the notion that MS in males is characterized by a more progressive disease course than in females.

Remyelination is a frequent phenomenon in acute or early MS lesions (145–147), as well as in chronic lesions (148). Overall, remyelination is an innate repair function of the nervous system as an effort to restore function to previously demyelinated nerve fibers (149). In MS, natural remyelination becomes impaired over time, possibly resulting in worse disease progression due to the inability to compensate for the loss of myelin and oligodendrocytes. In patients with MS, slightly more pronounced remyelination is observed in women compared with men (93), an outcome possibly linked to the differential effects of sex hormones on both the oligodendrocyte lineage and the neuroinflammatory processes (150). These findings, however, should be taken with caution, as the data highly relates to disease duration and usually does not reflect the initial remyelination capacity in male and female patients.

Concerning axonal damage, evidence was found of substantially higher nerve fiber density reductions in spinal pyramidal tracts of male MS patients compared to females (92). In this study, the nerve fiber density was reduced by 41% at C3 and 42% at T2 in men with MS and only by 19% at both C3 and T2 in women with MS (92). On the other hand, another study in brain tissue investigating the number of amyloid precursor protein (APP)-positive axons as a marker of acute axonal damage, did not detect any significant variation between sexes (151). However, findings from both these studies have to be treated with caution because of the small numbers involved.

Sex dimorphism in the CNS of MS patients likely influences lesion pathogenesis, and consequently, differences in the prevalence, and course of the disease. Sex dimorphism in CNS structure and physiology reflect the effects of hormone/receptor interactions within the CNS resident cells. Previously, it has been shown that sex steroidogenic enzymes' expression, as well as sex hormone receptors signaling, are enhanced in MS lesions and normal-appearing WM (NAWM) in a sex-specific way (152). In male MS lesions, estrogen synthesis, and estrogen receptor beta (ERβ)-mediated signaling are induced, whereas female MS lesions tend to show an increased progestogen synthesis (152). Therefore, the balance between local production of progesterone, estrogen, and androgen seems to differ between sexes, particularly in the lesional tissue, possibly contributing to sex differences in lesion development (152). From rodent studies, there is evidence that the three groups of sex hormones reduce neuroinflammatory and neurodegenerative processes and promote remyelination (116, 153–155). Progesterone, estrogen, and androgen signaling pathways consequently may represent an endogenous coping mechanism to counteract inflammation, demyelination, and neurodegeneration in MS, although with different degrees of protection due to a dimorphic modulation in males and females.

### Sex-Related Differences in Immune Responsiveness

MS pathology includes focal inflammatory demyelination with axonal damage (2, 156). Early in the disease course, RRMS is characterized by repeated inflammatory attacks in the CNS, resulting in localized damage or lesions in the brain and spinal cord. These lesions comprise T and B cells, macrophages, activated microglial cells, and other inflammatory cells (144). The more sustained immune response generally observed in women plays a role in the phenotypic differences of MS between sexes. As aforementioned, women are more likely to develop a relapsing MS disease course, synonymous with inflammation and demyelinating lesions (16, 32, 33). In contrast, men with MS develop, on average, a lower number of inflammatory lesions in the CNS (33, 36), but a higher number of degenerative lesions with extensive axonal loss (18, 47, 51, 157).

#### Sexual Dimorphism of the Peripheral Immune System

Several studies have shown differences in immune system activity between males and females (16, 19), a factor that may affect the development and progression of inflammatory diseases like MS. Generally speaking, females have stronger immune responses than males: females show on average a stronger humoral response (19, 94, 95), with a higher production of immunoglobulins (Igs), both IgM and IgG (96, 97), and a higher antibody response following immunization (19, 95). Females are also characterized by a more vigorous cell-mediated immune response (94, 95, 98), with increased T cell proliferation and activation (99). Both in animal models and humans, females are more likely to develop Th1 proinflammatory responses (95, 98–102), except during pregnancy when the Th2 anti-inflammatory response is predominant (100, 101, 158). In general, the ratio of CD4^+^ to CD8^+^ T cells is higher in females than in males (103, 104), with the relative number of circulating CD8^+^ T cells being significantly lower in the female population (100, 104). These sex-based differences that favor an increase in CD4^+^ T cells may provide a partial explanation for the stronger immune response to exogenous antigens, as observed in females.

Studies have also demonstrated naturally occurring sex differences in the distribution of peripheral immune cells. While age-linked variations in lymphocyte subsets have been reported between males and females (159), the total number of lymphocytes is similar. Nevertheless, on average, males have a decreased number of T cells (160), and post-menopausal women have lower numbers of both B and Th cells (161, 162). A significant difference was also detected in the percentage of regulatory T cells (Tregs), with higher percentages reported in a male vs. female, healthy population (163–165). Tregs in the male population not only show increased frequencies in the peripheral blood but also exhibit an enhanced suppressive and anti-inflammatory capacity compared to Treg cells from female donors (165). Lymphocyte subset enumeration in healthy controls also revealed higher B cells in females (166).

Finally, recent findings indicate a sexual dimorphism in the activation and regulation of a broad spectrum of peripheral cytokines and cytokine pathways. Several studies, for example, have described a more robust monocyte-derived cytokine production upon *ex vivo* stimulation with lipopolysaccharides (LPS) in men than in women (167–169), although others have found no sex differences (170, 171). Studies in mice also report that cytokine response of CD4^+^ T cells generally varies between males and females, with females displaying the highest Th1, Th2, and Tregs responses, contingent upon the stage of infection or type of antigen encountered (172–175). Evidence for differential cytokine expression in T cell subsets in response to antigen presentation by B cells and macrophages has also been demonstrated (176).

#### Sex Dimorphism of the Neuroimmune System

Biological sex plays an essential role in the normal physiology of the neuroimmune system as well as in the CNS response to inflammation. For example, sex differences are evident in microglia, the nervous system's primary resident immune cells. Although data in humans are still missing, recent studies (106, 107, 177) confirmed earlier research (178–180) showing differences in the structure, function, activation pattern, transcriptomic, and proteomic profiles in microglia from male and female mouse brains, thereby suggesting that microglia behave very differently in the two sexes. In a recent study, microglia cells were shown to be larger and more numerous in the CNS of healthy male mice compared with female mice (106). Transcriptomic and proteomic profiles of microglia also discriminated between male and female brains. Importantly differences were reflected in cell behavior, with male microglia presenting a higher antigen-presenting capacity and a higher potential to respond to CNS injury stimulants such as adenosine triphosphate (ATP) (106).

Similarly, in a second study, healthy male microglia had increased phagocytic activity and higher reactive oxygen species (ROS) levels (177). After a moderate-to-severe traumatic brain injury, microglial activation was shown to be more rapid and pronounced in males with a prominent activation of a highly proinflammatory phenotype and a rapid anti-inflammatory response. In contrast, a slower and less robust microglial activation was observed in females, also presenting with a less inflammatory phenotype and a delayed anti-inflammatory response (107). These findings suggest that microglia are more active in males and respond more vigorously to CNS injury. As a consequence of their higher state of alertness, male microglia may be worse at protecting themselves against CNS injury because they react more quickly to trigger their cell death program, thereby boosting the neurodegenerative processes in males compared to females. This may also apply to MS, a disease in which the activation of microglia likely plays an essential role in the effector phase of myelin breakdown and lesions formation (181, 182).

There are other immunocompetent cells in the CNS compartment, including astrocytes, and to a lesser extent, mast cells, myeloid cells, T and B lymphocytes. Although astrocytes appear to be sexually differentiated in many brain regions (183), the functional significance of these sex differences for normal brain and immune function remains an important area of future inquiry. Similarly, studies on CNS-resident dendritic and mast cells have been performed only in males thus far (183), so it remains to be determined whether there are sex differences in cell numbers or phenotype. No information has also been acquired concerning CNS-resident B cells. Sex differences were reported related to the activation and infiltration in the CNS of proinflammatory myeloid cells, monocytes, and macrophages, following a moderate to severe controlled cortical impact (CCI). In CCI male mice, a significant influx of peripheral myeloid cells was followed by a sustained proliferation of microglia. In contrast, myeloid infiltration and microglial activation were substantially lower in female CCI mice (177).

Sex differences in T cell trafficking into the CNS (184), as well as the number of CNS-resident T cells (185), were also reported. Sex dimorphism in T cells trafficking from the periphery to the CNS has been shown by using mice lacking mature T and B lymphocytes, i.e., RAG-1 knockout (KO) (186). In male and female RAG-1 KO mice, the adoptive transfer of CD3^+^ T cells led to substantially more stationary cells in the brain of the male compared to the female mice (184). This finding seems to contradict another, more recent study, that found aged female mice having almost twice as many CD4^+^ T cells in the CNS when compared to age-matched male rodents (185). However, in the latter study, age is a crucial factor that needs to be taken into consideration. Large numbers of CNS resident T cells in aged female brains suggest that older females are more likely to develop higher levels of inflammation and worse clinical outcomes following CNS damage. Accordingly, previous work has shown that both aged female mice and aged female rats exhibit more significant acute injury after ischemic stroke compared to aged males (187, 188). At present, it is still not clear whether ovarian hormones affect these sex-related effects, as all aged female rodents tested in these studies were post-menopausal, and young females were not investigated. However, another study from the same group suggested that, in aging females, T cell numbers in the injured brain negatively correlate with circulating levels of estradiol, ultimately suggesting that estrogen could suppress T cell trafficking into the CNS (189). These findings help to understand why post-menopausal women are at higher risk for developing progressive forms of MS (128) and overall neurodegenerative conditions (130).

Sex differences were also demonstrated in the activation and regulation of a broad network of cytokines. In the CNS, peripheral injections of lipopolysaccharides (LPS) resulted in the release of quantitatively and qualitatively distinct patterns of cytokines in male and female mice (108, 190). Sex-specific secretion of these cytokines was evident across several families of cytokines, including the GM-CSF/IL-3/IL-5 family, and the IL-2 family (also called the γ chain cytokine family), along with IL-10 and IL-13. Usually, males have a more robust production of cytokines such as CSF1 and CSF2, IFNγ, IL-10, with an overall stronger activation of the CXCL9 and CXCL10 pathways. In contrast, females have a higher release of IL-2, IL-15, CCL3, CCL5, IL-1α, IL-1β, and IL-4, with stronger activation of CSF3 and CXCL1 pathways (108, 190). Furthermore, females displayed a faster activation and subsequent resolution of the overall neuroimmune response, whereas males showed a slower but more persistent immune activation (108).

#### Sex Dimorphism of the Immune System in MS

Several investigators have examined sex differences in MS, revealing sexual dimorphism of the immune system on many levels. In peripheral blood, a sex bias toward peripheral proinflammatory Th1 responses to myelin proteins, e.g., increased IFNγ and decreased IL-5 levels, was described in women with MS compared to male patients (191, 192). Differently, men with MS show IL-5-skewed responses with low IFNγ (192). There was also evidence for sexual dimorphism in the peripheral proinflammatory cytokine response, as observed in a small cohort of RRMS patients. In this latter work, higher proinflammatory cytokine levels, such as TNFα, were detected in the males compared to the females (193). However, in another study, the peripheral cytokine profiles did not differ between sexes when considering all MS patients as a single group. Significant sex differences were instead described between disease subgroups (194). In RRMS patients, proinflammatory cytokine production was stronger in men than in women. In contrast, during the progressive phase of the disease, both SPMS and PPMS, cytokines levels were higher in females compared to males (194). Overall, these findings indicate that cytokine production and sex differences may differ between disease stages, being likely related to underlying disease mechanisms. However, linking these findings with the pathophysiology of each MS form is difficult, as peripheral cytokines levels may reflect either cause or effect, or a combination of the two, in the underlying pathological processes of MS.

Sexual dimorphism in MS also exists in the distribution of peripheral immune cells. In patients with clinically isolated syndrome (CIS), for example, women tend to present with a higher percentage of CD4^+^ Tregs, whereas men display higher levels of CD8^+^ Treg lymphocytes (104). The same pattern was found in the cerebrospinal fluid (CSF) (104).

A recent investigation provided interesting data regarding sex differences in IL-33 (112), a cytokine that modulates Th2 responses and decreases the differentiation of T cells into highly proinflammatory Th17 cells (195, 196). IL-33 expression is increased in NAWM and lesions of MS patients (197), implying that this cytokine may be part of a compensatory response to detrimental inflammation. Using the experimental allergic encephalomyelitis (EAE) model of MS, Russi et al. discovered that testosterone prompts meningeal mast cells to secrete IL-33 in the males, therefore blocking the development of Th17 immune cells (112). IL-33 expression was low in the lymph nodes, and CNS of healthy control mice and showed significant increases in expression in male but not female EAE mice (112). Since the male hormone testosterone directly induces IL-33 (112), the hypothesis to be still tested in humans is that with low levels of circulating testosterone, IL-33 is not adequately induced, thus leading to a predominant Th17 response in the absence of any detectable Th2 response. Reduced testosterone levels and a weak IL-33 response may also explain the increased MS and EAE susceptibility, as observed in aging males (99, 198).

In summary, in females, MS tends to provoke a greater Th1 and Th17 proinflammatory immune response, both in the peripheral blood and in the CNS. On the contrary, in males, MS causes the cells to adopt a Th2-type response and to suppress Th1/Th17 proinflammatory responses. Such a sexual dimorphism in the immune system of MS patients may explain the more inflammatory disease phenotype observed in the female compared to the male population.

### The Effect of Sex Hormones in MS

There is compelling evidence that sex hormones are essential in shaping the sex bias of the immune system and immune-mediated diseases, including MS (20, 21, 26, 113). Sex hormones such as estrogen, progesterone, prolactin, and testosterone have significant effects on both the immune and nervous systems, and most sex differences in MS may be revealed as a direct consequence of their actions.

According to several studies, low testosterone levels in patients with MS are linked to an increased risk of disability (49, 89). Therefore, testosterone therapy may slow disease progression and cognitive decline in men with MS (87). A second clinical observation on sex hormones in MS regards the effects of female hormones, namely estrogens, progesterone, and prolactin. High levels of estrogens and progesterone are protective in women with MS (118, 119), whereas higher levels of prolactin are generally associated with increased risk of developing the disease (199–202) and clinical relapses (201, 203), even though contrasting data have been published (204, 205).

#### Testosterone

**Figure 2:**
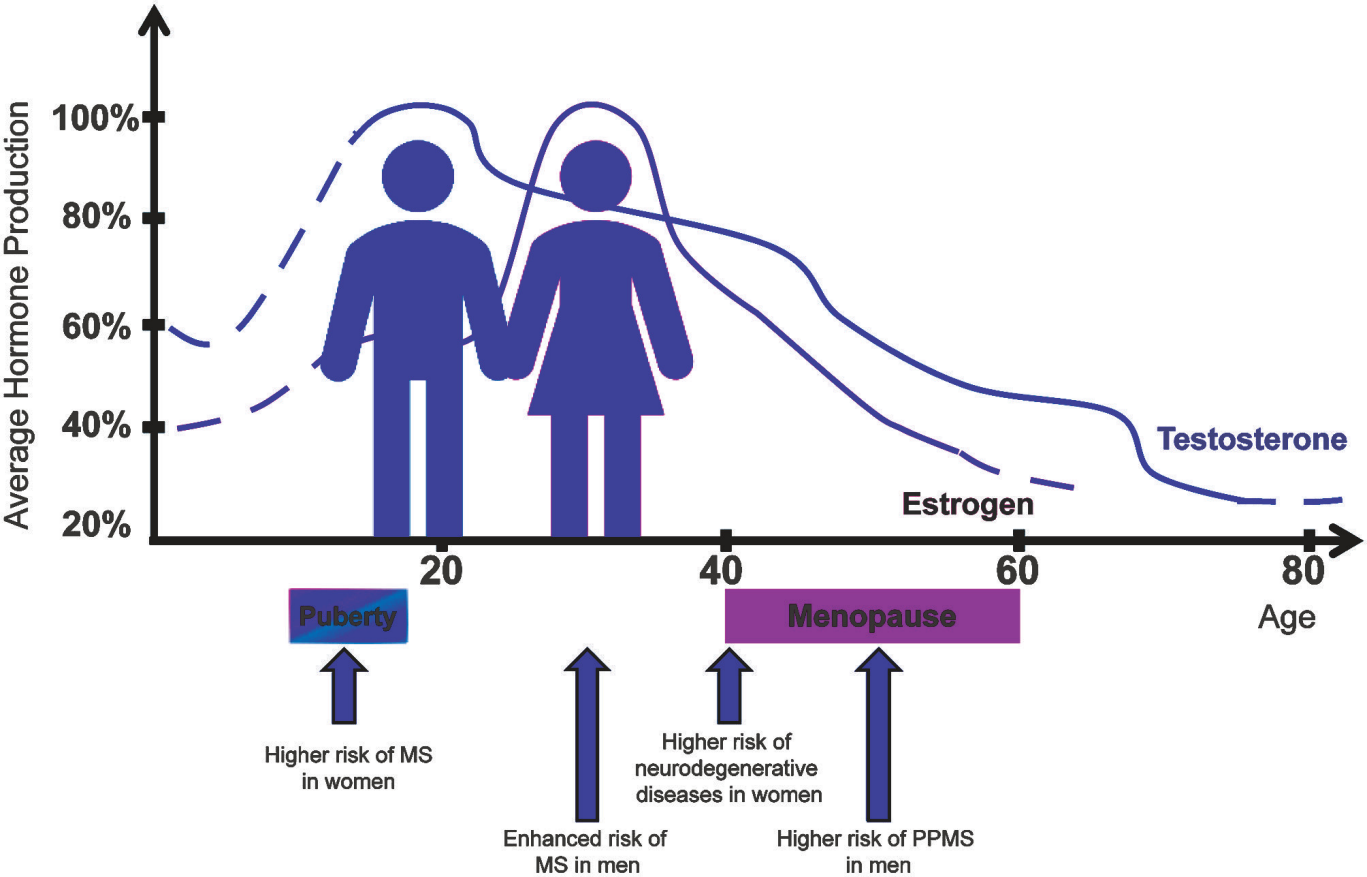
Sex hormone production in men and women in relation to MS risk by sex. The average percentage of estrogen (pink) and testosterone (blue) production from birth to age 80 years is indicated for female and male healthy individuals, respectively. Aging curves for estrogen and testosterone show a striking similarity to the MS incidence and clinical course. Hormone-related physiological conditions in women such as puberty and menopause exert significant influence both on disease prevalence and clinical outcomes. On the other hand, men are diagnosed with MS more frequently after puberty, just as their testosterone levels begin to drop.

Puberty, a time in life associated with increasing levels of sex steroids, is a pivotal time for MS and its sexual dimorphism (Figure 2). Sex bias observed in post-pubertal cases is absent in pre-pubertal cases, supporting the concept of puberty as a critical event for the dimorphic development of MS (127). Overall, puberty in females is associated with a higher risk of acquiring MS (206). In contrast, in males, the disease onset traditionally occurs later (age 30–40), i.e., at a time coinciding with the decline of the physiological levels of testosterone (111), the primary male hormone (Figure 2). Interestingly, the same phenomenon is observed in immune-mediated diseases other than MS. In rheumatoid arthritis, for example, the ratio of affected men in different age groups changes gradually, being four times higher in the older male population, namely men between 35 and 75 years of age (207).

Based on previous data showing that testosterone represents a natural anti-inflammatory hormone, exerting suppressive effects on both humoral and cellular immune responses (208), as well as the observation of a different prevalence of multiple forms of autoimmunity in the male population (19, 99, 113), it is likely that high levels of testosterone, usually detected in young men after puberty, may be protective against immune-mediated diseases. In MS, these high testosterone levels seem to mask an early disease onset, explaining the higher MS disease prevalence observed in older men, i.e., when the hormone levels tend to decrease physiologically.

Testosterone was also hypothesized to be protective in the sexually dimorphic development of MS (114, 115). Low testosterone levels associate with worse disability (49, 89) and more cognitive decline in MS patients (39, 89). Testosterone was shown to be neuroprotective in neuronal cultures (209), in MS animal models (116, 117, 210) and humans with MS (114, 115). In an *in vitro* system, for example, testosterone protected neuroblastoma cells from oxidative stress (209), whereas in the EAE model of MS, treatment with testosterone decreased clinical scores of disability, cognitive decline, inflammation, and demyelination (34, 109, 116, 210). Likewise, findings in two small clinical trials in men with RRMS suggest a neuroprotective effect of testosterone based on the measurement of improvements in the cognitive performance and slowing of brain atrophy, a finding which warrants further investigation (114, 115).

Testosterone effects in MS were tested in preclinical and clinical studies at doses within a safe and therapeutic range (Table 5).

**Table 5:** Hormonal dosages used in preclinical and clinical studies as well as routine clinical practice.

**Hormone**	**EAE/TMEV-IDD**	**MS**	**Clinical practice**
Progesterone	100–200 mg/14 or 60 days (pellet implant)	10–100 mg/day (oral route)	3–200 mg/day (Oral route)
Estradiol	2.5 mg/60 days (pellet implant)	0.02–0.04 mg/day (Oral route)	0.02–0.5 mg/day (Oral route)
Estriol	5 mg/60 day (pellet implant)	8 mg/day (oral route)	4–5 mg/day (Oral route)
Testosterone	100 mg/60 days (pellet implant)	100 mg/day (topical gel)	12.5–100 mg/day (topical gel)

#### Estrogens and Progesterone

**Figure 3:**
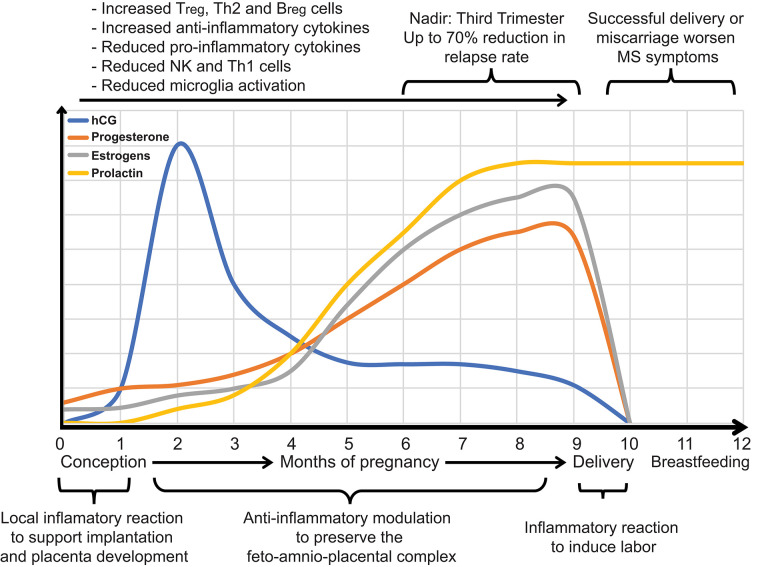
During pregnancy, it is evolutionarily advantageous for inflammatory immune responses that might lead to fetal rejection to be reduced and anti-inflammatory responses that promote the transfer of maternal antibodies to the fetus to be increased. Hormones modulate the immunological shift that occurs during pregnancy. Estrogens and progesterone increase throughout pregnancy and affect transcriptional signaling of inflammatory immune responses at the maternal-fetal interface and systemically. Levels of estrogen, progesterone, and human chorionic gonadotropin (hCG) throughout pregnancy are shown. Such alterations in the maternal hormonal and immune system ameliorate MS during pregnancy, especially during the third trimester, when hormones reach their peak. MS can flare up within a few months after giving birth.

Pregnancy, a physiological phenomenon typically correlated with sharp hormonal changes, profoundly affects the course of MS (Figure 3). Natural course studies in MS have shown that pregnancy positively affects the short-term course of the disease, being associated with up to a 70% reduction in relapse rates in the third trimester (81, 84, 125, 126). A marked resurgence of relapses is, in contrast, reported within the first 3 months after delivery (82, 132). Conversely, the long-term progression of MS is probably not influenced by pregnancy, since parous women with MS show no signs of increased disability over their lifetime compared with nulliparous women (132, 211–213). Pregnancy, however, may accelerate the rate of transition to SPMS (213), although a different study suggested that parous women with RRMS may be less likely to develop a progressive course of the disease (214).

The short-term protective effect of pregnancy on MS is most likely associated with an immunological shift that occurs during gestation. Indeed, during pregnancy, cell-mediated immunity is depressed to avoid fetal rejection (Figure 3), whereas humoral immune responses, promoting the transfer of maternal antibodies to the fetus, are increased (215–218). The primary hormones supporting pregnancy are estrogens and progesterone (219) that increase throughout gestation and alter the immune responses both systemically and at the maternal-fetal interface (215). This bidirectional interaction between hormones and the immune system contributes first to pregnancy outcomes, e.g., full-term or preterm birth, and spontaneous abortion, and secondly, to a lowered relapse rate in women with MS (Figure 3).

Estrogen levels, i.e., estradiol, estriol, and estrone rise through the entire pregnancy, peaking in the third trimester (Figure 3). Estrogen receptors act by regulating cells and pathways in the innate and adaptive immune system, also participating in the development of immune cells (220, 221). Physiological levels of estrogens, or the amount in birth control pills, do not seem to have significant effects on the immune system. However, at high pregnancy levels, estrogens suppress the activity of natural killer (NK) cells (222), inflammatory microglia (223), and Th1 cells as well as the production of proinflammatory cytokines (224, 225). Concurrently, estrogens increase the activity of Tregs, Th2 cells, and the production of anti-inflammatory cytokines (225, 226). Estrogens also have a significant impact on the development and function of B cells by triggering the expansion and activation of regulatory B cells (Breg), a specific subset of B lymphocytes with immunosuppressive functions (227).

Preclinical studies of MS confirmed that treatment with estrogens has anti-inflammatory properties. Estradiol, for example, was shown to yield a reduction in severity and frequency of two clinically different MS disease models, EAE (228–230) and Theiler's virus-induced demyelinating disease (TMEV-IDD) (231). Likewise, treating EAE mice with estriol protects them from disease activity (110, 232). Also, in a small phase 2, single-arm, crossover clinical trial of estriol treatment in women with RRMS, patients showed significant reductions in Gd-enhancing lesions as well as an increased expression of peripheral anti-inflammatory cytokines, e.g., IL-5 and IL-10, and a concurrent decrease of the proinflammatory TNFα (233, 234). In 2015, an additional study demonstrated that combined estrogen-progestin oral contraceptives, with high dose estrogens and in combination with IFNβ-1a therapy, significantly boost the overall anti-inflammatory activity of IFNβ in RRMS patients treated with the cytokine (235). Similarly, estriol, when added to glatiramer acetate, reduced relapses in a significantly broader cohort of women with RRMS (236).

Along with its immunomodulatory and anti-inflammatory roles via its effects on immune cells, estrogens also have neuroprotective effects (122–124). These effects are mainly supported by the observation that marked reduction in the circulating estrogens in women after menopause is associated with the development of neurodegenerative diseases such as Alzheimer's disease (AD) [Figure 2; (130)]. This concept is further reinforced by the finding of a reduced risk of AD and improved cognitive function in post-menopausal women treated with 17β-estradiol (237, 238). According to these observations, studies in MS showed that after menopause, the disease progresses more quickly (128, 129, 131), even though women have fewer relapses (129). As younger women with MS who underwent oophorectomy also found their disease getting worse after the procedure (128, 131), this worsening of MS in natural or induced post-menopausal women is likely linked to the dropping of estrogen levels. Thus, hormone replacement therapy, defined as the use of various types of estrogens alone or in conjunction with progestins, has been studied as a possible prophylactic against MS disease progression and neurodegeneration. Although treatment with systemic estrogen with or without progestin was associated with a better quality of life in post-menopausal women with MS (239), further studies are still necessary to investigate causality.

Progesterone, another female hormone, also has receptors on immune cells. The primary immune effects of progesterone are suppression of CD4^+^ T-cell differentiation, modulation of the Th1/Th2 balance, increase in Tregs production (240), and the downregulation of IFNγ and NK cells (241). Besides, progesterone has neuroprotective effects as it increases the proliferation of oligodendrocyte progenitor cells (OPCs) and promoting (re)myelination (153, 242).

Progesterone and synthetic progestins have been shown in animal models, i.e., EAE and the cuprizone model of demyelination, to diminish myelin damage, reduce clinical severity, modulate neuroinflammation, and partially reverse the age-dependent decline in remyelination (120, 121, 242, 243). Nevertheless, no protective effect was observed in women with MS, when progesterone was administered to prevent post-partum exacerbations (244).

Hormonal effects that are potentially clinically relevant in MS were adequately characterized in preclinical and clinical studies using doses up to the maximum tolerated dose (MTD) and within the dosing ranges to routine clinical practice and route of administration (Table 5).

#### Prolactin

Prolactin, also known as luteotropic hormone or luteotropin, is a hormone produced in the pituitary gland. Besides its primary roles in mammary gland development and lactation, prolactin is supposed to be involved in many alternative functions, including immune modulation promoting B cell maturation and autoreactivity (245), and cell proliferation boosting remyelination (205, 246).

The effect of prolactin on MS is disputed as some studies showed higher levels of this hormone in MS patients (199–202), especially during relapses (201, 203), while other studies have challenged this view (204, 205). Similar contrasting results have been found in preclinical studies. In the EAE model, for example, prolactin administered at a low dose did not exacerbate the disease when administered neither prophylactically nor therapeutically, but the high dose did (203). Also, bromocriptine, a prolactin suppressor, was shown to inhibit lymphocyte proliferation, implying that prolactin is detrimental in MS (247). Nevertheless, prolactin was also shown to induce the proliferation of OPCs, ultimately promoting (re)myelination (205). Overall, prolactin seems to exert a dual effect in MS and, therefore, at this time, it cannot be recommended as a therapeutic agent in MS.

#### Gender Identity Disorder and MS Susceptibility

Also known as gender dysphoria, gender identity disorder (GID) refers to a condition wherein patients have a significant level of discontent with their birth-assigned sex. Hormonal interventions are being increasingly used to treat people with GID, but the influence of the high-dose hormone treatments on MS risk and disease course remains largely uncharacterized.

In a recent study, it has been reported that men with GID under transgender hormone therapy of the male-to-female (MTF) type showed nearly 7-fold higher risk of developing MS compared to men without GID (248). In this male population, an altered balance of sex hormones, both constitutional and secondary to hormonal treatments, which generally comprises estrogen and anti-androgens along with sex reassignment surgery, likely increases MS risk. This finding supports the postulated link between low testosterone levels and MS susceptibility (49, 89, 113) and highlights a need for further research on the effects of feminizing sex hormones therapies on MS disease susceptibility.

The use of testosterone in female patients diagnosed with MS as a transgender hormone therapy of the female-to-male (FTM) type, also remains unclear. Testosterone dosage levels for cross-sex hormone therapy often differ in amount compared to those used in clinical trials (115, 117, 249). A previous case study presented a fully transitioned FTM transgender male with RRMS, in whom clinical exacerbations and progressive disability were still evident after surgical intervention and long-term testosterone therapy (250). However, it is still unclear whether steady doses of testosterone, both before and after FTM transition, aggravated disease activity, and precipitated disability progression.

### The Effect of Sex Chromosomes

Sexual dimorphism ultimately arises from sex chromosomes, either directly from sex-linked transcriptional products or secondarily from sex hormones produced after the differentiation of female- or male-specific reproductive tissues.

Genes encoded on the sex chromosomes initiate all biological sex differences. An essential gene on the Y chromosome is Sry for Sex-determining Region Y (251, 252). This gene induces the undifferentiated gonad to differentiate into testes rather than ovaries. Once formed, testes secrete distinct hormones, e.g., testosterone, that generate sex differences at diverse non-gonadal tissues, organs and systems, including the external genitalia, the immune system, the nervous system, the cardiovascular system, and the skeletal system (252).

Besides these hormonal and meta-hormonal determinants of sexual dimorphism, there are also direct genetic divergences arising from the difference in sex chromosomes complement that could also contribute to the phenotypic sexual dimorphism. An innovative mouse model system, known as Four Core Genotype (FCG), has been recently established to study the sex chromosomes effects without the confounding action of a specific gonadal type (251, 253). In this model, the Sry gene has been knocked out from the Y chromosome, resulting in Sry-deficient mice, i.e., XX and XY^−^ phenotypic gonadal females (XX^f^ and XY^f^). Further, the insertion of Sry as a transgene onto an autosome generates XX and XY phenotypic gonadal male mice (XX^m^ and XY^m^). Experiments in the FCG model allow for the testing of sex chromosome effects in two different sex-specific hormonal conditions, i.e., XX^f^ vs. XY^f^ and XX^m^ vs. XY^m^ (251, 253).

In a recent study in EAE (133), comparisons between XX and XY FCG mice uncovered a previously underestimated effect of sex chromosomes not confounded by gonadal hormones. Gonadectomized (Gdx) XX^m^ or XY^m^ mice had EAE induced with the myelin proteolipid protein peptide PLP_139−151_. Clinical signs and disease course were overall more severe in XX^m^ mice, as compared to XY^m^ mice (133). A similar difference in disease severity was evident after comparing Gdx XX^f^ vs. Gdx XY^f^ mice (133). To ascertain whether the observed sex chromosome effect in EAE was a consequence of the influence sex chromosome complement has on the immune system, authors adoptively transferred autoantigen-stimulated lymph node cells (LNCs) from Gdx and PLP_139−151_-immunized XX^f^ or XY^f^ mice into wild-type female mice. LNCs derived from Gdx XX^f^ mice, as compared with those collected from XY^f^ rodents, promoted the development of a more severe form of EAE (133). These observations demonstrate for the first time, sex chromosomes affect the induction of encephalitogenic immune responses following EAE immunization in mice. The authors also examined the underlying mechanisms by which the XX sex chromosome complement may promote the development of EAE, ultimately highlighting an augmented release of anti-inflammatory Th2 cytokines from cells derived from XY^f^ mice (133). Interestingly, Th2 cytokines, such as IL-13, IL-4, and IL-10, had previously been associated with reduced susceptibility and disease severity in EAE (254–256). As such, the enhanced Th2 cytokine activity in XY^f^ mice may protect from severe EAE as compared with XX^f^ mice. Overall, this study has provided the first evidence that the XX sex chromosome complement likely results in a greater susceptibility to MS. Sex chromosome divergences that may explain this sexual dimorphism include (1) the potentially double genomic dose of X genes in XX cells, (2) the presence of Y genes only in male cells, and (3) the presence of paternal genomic imprinting on the X chromosome arising only in females.

#### The X Chromosome

Typically, females inherit two copies of the X chromosome, one from each parent, whereas males inherit one maternal X chromosome and one paternal Y chromosome. To prevent females from having twice as many X-linked genes on their sex chromosomes as males, one of the X chromosomes is randomly inactivated during embryogenesis. Once an X chromosome becomes genetically inactive and untranscribed, it remains so throughout life. The process of X chromosome inactivation results in a phenomenon known as cellular mosaicism, which is most advantageous in females since it ameliorates the deleterious effects of X-linked mutations (23). However, there are some exceptions: most of the genes have homologous regions on the Y chromosome, and certain genes can escape X chromosome inactivation at variable frequencies (257). In this case, a female ends up with too many active copies of a particular gene. Considering the critical role played by the majority of the X chromosome gene products in the immune response, this phenomenon may be of relevance in terms of the over-reactive immune system and generally immune-mediated diseases.

A second molecular mechanism of X inactivation that could also contribute to the sex bias in immune-mediated diseases is called “skewed X chromosome inactivation.” A skewed X-inactivation happens when cells show a preferential inactivation of one X chromosome over the other, leading to an uneven number of cells with the same X chromosome inactivated (23). While there may be random X inactivation in most tissues, locally skewed X-inactivation may exist in the thymus leading to inadequate thymic deletion (24). T cells tolerized to self-antigens encoded by one of the two X chromosomes may be reactive to self-antigens encoded by the other X chromosome when encountered in peripheral tissues (24). This specific mechanism was correlated with some immune-mediated diseases with female predominances, such as autoimmune thyroid disease (ATD) and scleroderma (258, 259). Nevertheless, skewed X inactivation has not been found in the cases of other, more frequent, immune-mediated diseases, including lupus erythematosus systemic (LES) (260), type 1 diabetes (24), and MS (261, 262). Thus, currently, there is still too much conflicting evidence of an association between an increased frequency of skewed X-chromosome inactivation and diseases driven by the immune system.

The X chromosome is known to carry the highest number of immune-related genes and has been implicated in sex differences in immune-mediated diseases (257). Thus, the X chromosome has become a research topic of great interest, and many studies recently sought to understand the role of X-linked genes in the development and progression of MS and generally immune-mediated diseases (257). Notably, three X chromosome candidate genes have been at the forefront of genetic association studies in MS: PLP (located on Xq22) (134), cytochrome b-245 β chain (CYBB or NOX2, Xp21) (263), and gamma-aminobutyric acid ionotropic type A receptor (GABRA3, Xq28) (135). Although these studies did produce some evidence for association with MS, none of these genes was actually identified in genome-wide association studies (GWAS) (264, 265). It should be noted, however, that the X chromosome is usually excluded from GWAS analyses despite being assayed on all current GWAS platforms. The most recent and possibly more extensive meta-analysis on genome-wide MS data to date (266), identifies a total of 233 loci significantly associated with MS, only one being mapped on the X chromosome. This may sound suspicious, given the strong sex-specific component of the disease. However, the lack of association between the X chromosome and complex genetic traits is not uncommon. Although the X chromosome contains only 5% of all human genes, almost 10% of Mendelian disorders have been assigned to the X chromosome (267). Yet, only 0.5% of the associations identified by GWAS have been ultimately reported on the X chromosome (268, 269). Several different reasons may explain this lag in X chromosome GWAS findings, including the omission of the X chromosome from most GWAS analyses and the lack of specific pipelines for X-wide (XWAS) association studies.

More recently, a fourth X-linked gene has been associated with MS (136). UTX [ubiquitously transcribed X chromosome tetratricopeptide repeat protein, also known as Kdm6a lysine (K)-specific demethylase 6A] is a gene that encodes a protein that functions in the catalysis of the demethylation of tri/dimethylated histone H3. UTX is heavily implicated in modulating a broad range of immune responses such as the proinflammatory response of macrophages (270), Th cell differentiation, and indirectly the maturation of IgG-secreting plasma cells (271). UTX is an X-linked gene with evidence of homologous regions on the Y chromosome, and, therefore, it escapes from X inactivation (257), likely contributing to higher UTX expression in the female population relative to the male population (272). A recent study examined the effect of UTX on EAE, specifically focusing on mice lacking the UTX gene in their CD4^+^ T cells (136). These mice had reduced clinical symptoms compared with mice with intact UTX. There was evidence of reduced inflammation and axonal damage in the spinal cord, therefore suggesting the deletion or inhibition of the UTX gene has a protective effect in EAE and possibly in MS. To date, however, UTX was not associated to MS in any GWAS analysis, possibly because of the lack of studies focusing on the X chromosome.

Another study involving the X chromosome, a cytogenetic analysis in patients with MS, identified abnormal X chromosomes in 50% of the study cohort. Abnormalities were premature centromere division and structural aberrations that could imply a preferential clustering of chromosomal breakpoints (139). Correlation between clinical and cytogenetic data also showed that cytogenetic abnormalities were prevalent in patients with a high relapse frequency or with progressive forms of the disease (139). To our knowledge, such cytogenetic analyses have not been repeated in subsequent studies. It is therefore not clear what the significance of these findings may be, nor whether these chromosomal aberrations directly cause the disease instead of being just a consequence of the disease activity.

#### The Y Chromosome

Fewer studies have analyzed the role of the Y chromosome in MS. Y chromosomes have long been characterized as “genetic wastelands,” whose primary role was to trigger the development of the male sex. This view has been challenged in recent years with the identification of a new unforeseen association between the Y chromosome and the immune system (140). Similar to the X chromosome, also the Y chromosome seems to significantly influence inflammatory responses in males, resulting in genetic susceptibility or protection to complex immune-mediated diseases (140). Notably, the Ubiquitously Transcribed Tetratricopeptide Repeat Containing, Y-Linked (UTY) gene appears to be a promising candidate underlying the association between the Y-chromosome and the immune-related susceptibility to diseases like MS. Interestingly, UTY is the Y chromosome homolog of the UTX gene mentioned above, which was recently linked to EAE and possibly MS (136).

Further support for the Y chromosome as a potential dysregulated immunity locus derives from studies in animal disease models. In EAE, for example, experiments on consomic strains of rodents demonstrated that the Y chromosome heavily influences the susceptibility to EAE as well as its severity (141). Also, the transcriptomic analysis performed in macrophages and CD4^+^ T cells revealed a large number of differentially expressed Y-chromosome genes, when more susceptible mouse strains were compared to less susceptible strains (141). Data were confirmed in humans by an analysis of the CD4^+^ T cell transcriptome in male patients with CIS vs. healthy male donors (141). A large proportion of the same Y-linked genes identified in the mouse model were showed differentially expressed in CD4^+^ T cells from the CIS patients vs. healthy controls, providing further evidence for an evolutionarily conserved mechanism of gene regulation by the Y chromosome (141).

## Conclusions

It is widely recognized that understanding sex differences in diseases is essential for discovering sex-biasing factors that predispose or protect from diseases and for developing optimal monitoring strategies and therapies for women and men. Accordingly, the National Institutes of Health (NIH) has acknowledged the critical implications of sex differences in science (273), ultimately implementing, in 2016, new guidelines for NIH grants that prevent sex bias in basic, preclinical, and clinical research (274).

Sex-specific medicine is aimed at considering the individual characteristics of male and female biology, taking into equal consideration the interest of both sexes. Thus, women should receive more considerable attention when specific data on women's health is lacking, while men should receive more attention when data on men's health is lacking. For example, more data on women may be needed regarding RRMS as women outnumber men three to one in this form of the disease. In contrast, more data on men are urgently needed in progressive MS since male sex is associated with faster and worse disease progression. Nevertheless, despite our knowledge of the differences between males and females, there is still no sex-specific health care in MS, and the prevention, management, and treatment do not reflect the most evident and essential risk factor for patients, i.e., biological sex. This omission is delaying a more efficient health care system, as sex-specific therapies and monitoring strategies may be more effective than a “standard of care” determined by averaging responses across large cohorts of patients and would equally benefit patients of both sexes.

MS exhibits an evident sexual dimorphism in both disease susceptibility and progression: MS is more common in women, but the severity of the disease course is worse in men (36, 39, 57, 58). The increased incidence of the disease among women has been widely explored, with differences between male and female attributed to the more active immune system, e.g., higher numbers and stronger proliferative capacity of circulating T-cells (16, 95, 98, 102), stronger cellular immune responses to antigen (95, 98), as well as higher levels of B cells (166), and circulating antibodies (95, 105), observed in females compared to males. Such a stronger peripheral immune response of females may explain why women with MS display a more inflammatory phenotype of the disease, as characterized by a higher number of inflammatory exacerbations and Gd-enhancing lesions on MRI.

Conversely, it is still unclear, why men have a faster and worse disability progression, despite their “weaker” peripheral inflammatory responses. Generally, the gradual accumulation of disability in MS patients results from a variety of mechanisms, including inflammatory reactions confined to the CNS such as leukocytes infiltrates contributing to demyelination and axonal/neuronal damage (275, 276), microglial activation associated with the development of cortical lesions (277), and other complex immune responses resulting in inflammatory secretory products in the CSF space (278). Interestingly, inflammatory responses in the CNS appear more rapid and pronounced in males. Female and male microglial cells, for example, display differences in the structure, function, transcriptomic, and proteomic profiles, and responses to CNS injury (106, 107). Overall, basal male microglia were shown to have a higher antigen-presenting capacity, as well as a higher potential to respond to CNS injury stimuli (106). In parallel, CNS injury triggers a rapid and substantial microglia activation in males with a more inflammatory phenotype that produced a rapid, single-phase, and sustained peak. In contrast, CNS injury triggers a less robust microglia phenotype in females with biphasic proinflammatory response peaks, and a delayed anti-inflammatory peak (107). Altogether these observations challenge the paradigm that females always have stronger immune responses than males by suggesting that male patients with MS have quantitatively higher amounts of CNS inflammation compared to their female counterparts and providing a causal explanation of the worse clinical outcome observed in males compared to females.

Historically, it was argued that sex differences in MS are primarily due to sex hormones. Nonetheless, emerging evidence demonstrates that sex differences may also be mediated by mechanisms other than hormones, and in particular, by X and/or Y chromosome gene products (134–139, 141, 261, 262). Possibly, genes on the X and Y chromosomes contribute to MS susceptibility and progression in a polygenic fashion. However, no X and/or Y specific chromosome loci have yet been identified in MS.

## Author Contributions

FG conceived and designed the entire review and wrote the paper. KD and AP reviewed and edited the manuscript. All authors read and approved the manuscript.

## Conflict of Interest

The authors declare that the research was conducted in the absence of any commercial or financial relationships that could be construed as a potential conflict of interest.
